# The Prevalence of Knee Osteoarthritis and Its Association With Obesity Among Individuals in Saudi Arabia

**DOI:** 10.7759/cureus.49625

**Published:** 2023-11-29

**Authors:** Albaraa A Altowijri, Aseel A Alnadawi, Jawaher N Almutairi, Alhanouf K Almutairi, Meshari S Alhawiti, Ahmed K Abu Sinah, Joud A Alhassun

**Affiliations:** 1 College of Medicine, University of Tabuk, Tabuk, SAU; 2 College of Medicine, Unaizah College of Medicine and Medical Sciences, Qassim University, Unaizah, SAU; 3 College of Medicine, King Faisal University, Hofuf, SAU

**Keywords:** body mass index: bmi, obesity, osteoarthritis (oa), knee, prevalence

## Abstract

Introduction

This research, set in 2023 in Saudi Arabia, addresses the rising prevalence of knee osteoarthritis (OA) among obese individuals. It explores associations with age, BMI, and gender, aiming to enhance our understanding of this pressing health issue within the Saudi context and contribute to global research on OA and obesity.

Methodology

A retrospective cohort study was conducted at King Saud Hospital and Buraydah Central Hospital in the Al-Qassim region from November 2022 to February 2023. It included the patients’ records that fit the inclusion criteria during the study period. A data collection sheet was used to collect data, and data was analyzed using SPSS Statistics version 27.0.1 (IBM Corp. Released 2020. IBM SPSS Statistics for Windows, Version 27.0. Armonk, NY: IBM Corp.).

Results

In 2023, a study on knee OA among 267 obese individuals in Saudi Arabia yielded significant findings. The median age of participants was 60 years, with an average weight of 77.00 kg (IQR:15) and a median BMI of 29.14, categorizing participants as overweight. Ninety percent (n=240) of knee OA cases occurred in individuals classified as “obese” (n=118) or “overweight (n=122)”. Furthermore, OA primarily affected both knees in 79.4% (n=212) of cases.

Conclusion

Study participants, predominantly older and female, reflect OA’s age-related and gender-specific prevalence trends. Notably, excess weight and a higher BMI highlight the role of obesity as a significant OA risk factor. Bilateral knee involvement is common, with a strong link between OA and obesity.

## Introduction

Osteoarthritis (OA) is the most prevalent type of arthritis in the United States (US), which is also one of the main causes of disability. Symptoms are taken into consideration together with radiographic results when defining them in epidemiologic studies [1]. In terms of pathophysiology, joint margins and subchondral regions develop reactive new bone as a result of OA, which also causes the cartilage to deteriorate in the joints. Most frequently in the distal and proximal interphalangeal joints but also in the hip and knee joints, this degradation is caused by a breakdown of chondrocyte function. Recent research has shown that OA affects the entire joint, not only the articular cartilage [2].

Among US individuals, the prevalence of clinical OA has increased. There have been some published studies that support this rise as a result of population aging and obesity [1]. In Rotterdam County, Netherlands, a study conducted in 2007 among people aged more than or equal to 55 years showed how OA of the knee and hip is the primary cause of disability in the elderly and how its prevalence will rise as Western culture becomes older [3]. Population-based case-control research conducted in 2001 in three England health districts (Southampton, Portsmouth, and North Staffordshire) revealed a significant link between obesity and knee OA [4].

According to one study, the probability of developing symptomatic knee OA over the course of one's lifetime is around 40% for men and 47% for women, with higher risks among obese people [1]. Moreover, the study proposed that a higher prevalence and more severe OA are related to females more than males [1]. Even though the precise process is unknown, the biggest risk factor for OA is advanced age. It is most likely linked to various changes in joint tissue's ability to adjust to biomechanical stresses due to the accumulation of a significant number of risk factors over time [1].

Obesity has long been known to increase the risk of developing OA, particularly knee OA [5]. Numerous studies have demonstrated that having a higher body weight increases the likelihood of developing OA, regardless of the exact mechanisms [2].

A systematic review study performed in France at Clermont University in 2016 showed that the most likely way in which obesity causes OA is by increasing the mechanical pressures that are placed on the hip and knee joints when engaging in physical activity. Also, each extra kilogram of body weight produces 6 kg of stress on each of the two knees. The increased mechanical stress on weight-bearing joints leads to cartilage degeneration [6].

Several factors contribute to the higher incidence of OA in obese patients [7]. These factors include a large volume of fat mass and hyperexpression of pro-inflammatory mediators (cytokines, adipokines). As a result, there will be a high mechanical load on the joints as well as the development of low-level metabolic inflammation [7].

Conservative treatment such as weight loss can modify obesity, and its potential importance in decreasing the incidence of OA cannot be underestimated [8]. While we were searching about this topic, we noticed insufficient studies in Saudi Arabia that have been conducted to determine the prevalence and association of knee OA among obese individuals. The aim of this study is to test the hypothesis that there is a significant positive correlation between osteoarthritis and obesity, see the impact of factors such as age, BMI, and gender, and see if environmental factors could be a risk. Furthermore, this study aims to expand the knowledge of this problem in our area and help establish baseline data in the region.

## Materials and methods

In this quantitative observational retrospective cohort study, we examined the prevalence of knee OA and its association with obese individuals in Saudi Arabia. The study was carried out from August 2022 to August 2023 at the Orthopedic Department of King Saud Hospital and Buraydah Central Hospital, Al Qassim Region, Saudi Arabia. We calculated the sample size at a 95% confidence level by using OpenEpi, version 3, an open-source calculator-SSPropor (OpenEpi, www.OpenEpi.com).

During this period, we collected data from the medical records of patients who were diagnosed with knee OA at the outpatient clinics in these hospitals. To ensure a representative sample, we included all eligible medical records without performing a sample size calculation. The inclusion criteria encompassed Saudi women and men 40 years of age and older who were diagnosed with knee OA.

Data collection was conducted using a structured data collection sheet, which included age, gender, residency, weight, height, BMI, date of diagnosis, and site of the affected knee. Before initiating the main study, a pilot study was conducted to test the feasibility, reliability, and clarity of the data collection process. This pilot study involved a sample of records from King Saud Hospital and Buraydah Central Hospital.

For statistical analysis, the sample size was 267 participants. The analysis involved both descriptive and inferential statistical tests. Descriptive statistics were used to summarize and describe the characteristics of the study participants. Frequencies and percentages were calculated for categorical variables. Mean and standard deviation were computed for normally distributed continuous variables such as age, height, weight, and BMI, whereas median and interquartile range (IQR) were determined for non-normally distributed continuous variables. The normality of the data was checked by the Shapiro-Wilk test.

For independent samples, the Kruskal-Wallis test was used to see the difference in the site of knee OA and different demographic variables due to the non-normal distribution of data. Additionally, Fisher's exact test was conducted to find an association between the demographic characteristics and the site of knee OA. The significance level for all statistical tests was set at p<0.05, indicating a 95% confidence interval. All statistical calculations were performed using SPSS Statistics version 27.0.1 (IBM Corp. Released 2020. IBM SPSS Statistics for Windows, Version 27.0. Armonk, NY: IBM Corp).

## Results

The sociodemographic characteristics are presented in Table 1. The median age of the participants was 60 years. The participants' median weight was 77.00 kg, with an interquartile range of 15 kg, and the median height was 160 cm. The median BMI of 29.14 falls within the overweight category. The gender showed that the majority of participants were female (72.7%) (Table 1).

**Table 1:** Sociodemographic characteristics of participants having knee OA BMI: body mass index, SD: standard deviation, IQR: interquartile range

		Mean/median	SD/IQR (Q_3-_Q_1_)	Count	%
Age		60	13 (66-53)	-	-
Weight		77.00	15 (85-70)	-	-
Height		160	10 (165-155)	-	-
BMI		29.14	(33.00-26.83)	-	-
Gender	Female	-	-	194	72.7%
	Male	-	-	73	27.3%
Residency	Saudi	-	-	267	100.0%

**Figure 1:**
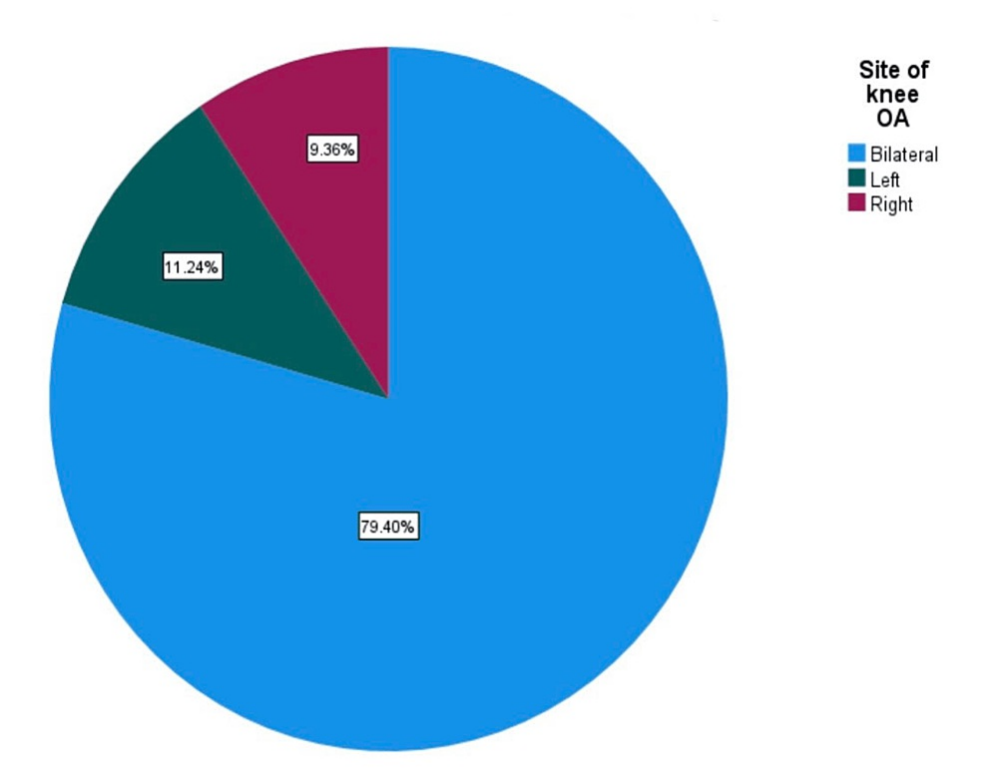
Prevalent site of knee OA OA: osteoarthritis

**Figure 2:**
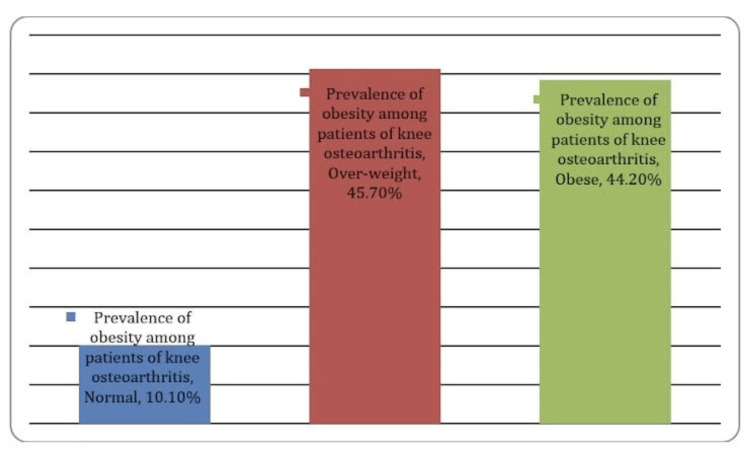
Prevalence of obesity among patients with knee OA

Table 2 shows that knee OA primarily affects both knees (bilateral involvement) in the majority of participants, accounting for 79.4%. Additionally, there was a strong association between knee OA and obesity, with 90% of cases occurring in individuals who were obese and overweight (Table 2, Figures 1-2).

**Table 2:** Prevalent site of knee OA and its association with obesity OA: osteoarthritis, BMI: body mass index

		Count	%
Site of knee OA	Bilateral	212	79.4%
	Left	30	11.2%
	Right	25	9.4%
BMI	Normal	27	10.1%
	Obese	118	44.2%
	Overweight	122	45.7%

**Figure 3:**
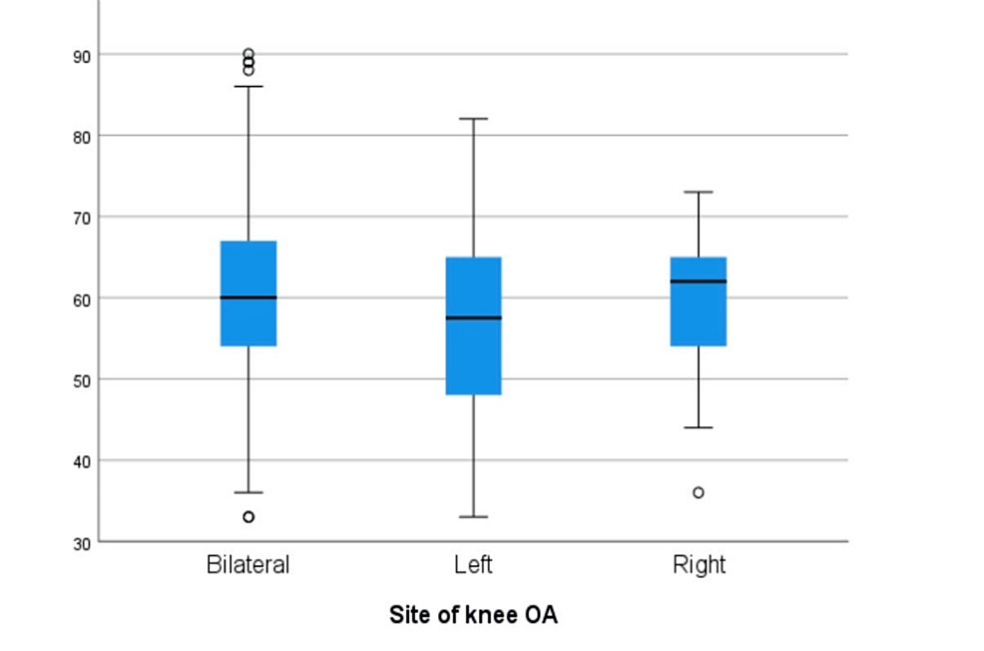
Age distribution at three different sites of knee OA

**Figure 4:**
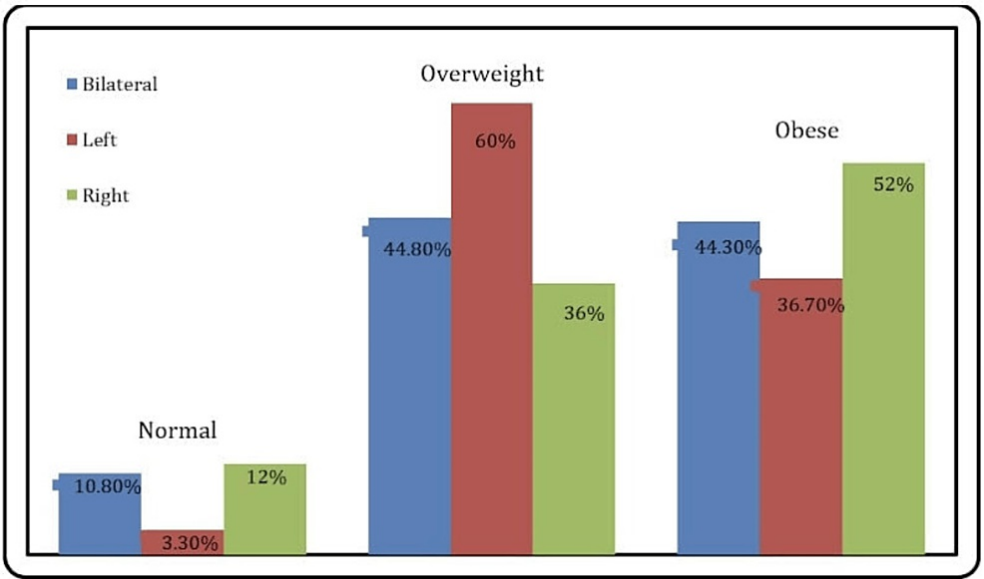
Site of knee OA by BMI class OA: osteoarthritis, BMI: body mass index

The analysis of sociodemographic variables in relation to the site of knee OA provides valuable insights into the complexity of this condition within the studied population. While knee OA is a multifaceted ailment influenced by various factors, this specific analysis did not reveal significant associations between several key sociodemographic variables and the site of OA manifestation. Firstly, age did not exhibit a significant difference among those with bilateral, left, or right knee OA. Secondly, BMI also failed to show significant differences among the OA sites (Table 3, Figures 3-4).

**Table 3:** Association between sociodemographic variables and the site of knee OA * Kruskal-Wallis for independent samples, ** Fisher's exact test, BMI: body mass index, IQR: interquartile range

		Site of knee OA						p-value
		Bilateral		Left		Right		
		Median	IQR (Q_3-_Q_1_)	Median	IQR (Q_3-_Q_1_)	Median	IQR (Q_3-_Q_1_)	
Age		60	13 (67-54)	58	17 (65-48)	62	11 (65-54)	0.292*
Height		160	10 (165-155)	165	12 (169-157)	159	9 (165-156)	0.267*
Weight		77	17 (85.00-68.00)	77	13 (85 -72)	77	15( 85-70)	0.727*
BMI		29.19	3.16 (33.06-29.90)	28.29	(33.04-26.83)	30.11	4.23 (31.25-27.02)	0.992*
Gender	Female	155	73.10%	19	63.30%	20	80.00%	0.365**
	Male	57	26.90%	11	36.70%	5	20.00%	
BMI class	Normal	23	10.80%	1	3.30%	3	12.00%	0.380**
	Obese	94	44.30%	11	36.70%	13	52.00%	
	Overweight	95	44.80%	18	60.00%	9	36.00%	

## Discussion

Our study aimed to determine the prevalence and association of knee OA in Saudi Arabia in 2023. Regarding sociodemographics, the median age of 60 years among the participants indicates that knee OA predominantly affects middle-aged to older individuals [9]. The average weight of 77.00 kg, with a notable interquartile range, suggests considerable variation in weight within the studied population. Higher weight is a well-established risk factor for knee OA, indicating that individuals with higher weight levels are at increased risk [10]. The median BMI of 29.14 falling within the overweight category further emphasizes the potential risk within this group, underscoring the importance of weight management in OA prevention [11].

A significant gender disparity was found in our study, with a majority of female participants (72.7%). This finding aligns with existing research indicating that knee OA is more common in women, especially as they age [12]. It highlights the need for gender-specific considerations in the prevention and management of knee OA.

Our study indicates that knee OA predominantly affects both knees (bilateral involvement) in a significant majority of participants. This suggests that OA often manifests simultaneously in both knees within this population. This could have implications for treatment strategies, emphasizing the need for comprehensive management of both knees when dealing with knee OA cases. Messier et al. (2008), which examined knee OA prevalence among obese individuals, observed that bilateral involvement was also common [13].

The study establishes a clear association between knee OA and obesity. An overwhelming 90% of the cases occurred in individuals classified as “obese” or “overweight.” This underscores the significant role of obesity as a risk factor for knee OA in this population. Obese individuals should be a primary focus for preventive measures and interventions to reduce the burden of knee OA. The association between knee OA and obesity remained consistent, with the majority of cases occurring in individuals classified as obese or overweight [14,15].

Our study did not find significant differences in age among those with bilateral, left, or right knee OA. This suggests that age may not be the primary determinant of the specific site of OA onset in this particular population. These findings emphasize the complexity and heterogeneity of OA presentations and indicate that other factors might play a more prominent role in determining the sight of OA. The lack of significant differences in BMI among the different OA sites contradicts the widely recognized relationship between obesity and knee OA [16]. It suggests that, within this specific population, factors other than BMI may have a stronger influence on the localization of knee OA. There were no significant associations between height, weight, gender, and BMI class and OA site. These results further support the notion that, in this particular group of obese individuals in Saudi Arabia, sociodemographic characteristics do not strongly dictate the specific site of knee OA.

In a previous study conducted by Shane Anderson et al. (2010), which examined the sociodemographic factors influencing knee OA sites among obese individuals in a different population, it was observed that age did play a significant role, with older individuals more likely to develop bilateral OA [17]. However, the relationship between BMI and OA site varied compared to our current study, with a stronger association between higher BMI and bilateral OA [18,19]. These differences underscore the importance of considering regional and population-specific factors in understanding the complex interplay between sociodemographic variables and knee OA sites.

While this study provides valuable insights into the prevalence and association of knee OA among obese individuals in Saudi Arabia, it is essential to acknowledge its limitations. Firstly, the cross-sectional design of the study limits our ability to establish causality between obesity and knee OA. Longitudinal studies would be necessary to confirm the temporal relationship between these variables. Secondly, the data did not consider other potential factors, such as physical activity levels, dietary habits, or genetic predispositions, which could contribute to knee OA development. Finally, the study’s reliance on self-reported data may introduce recall bias and underestimate the true prevalence of knee OA.

## Conclusions

This study provides valuable insights into knee OA and its sociodemographic associations. The participants, predominantly older and female, reflect OA's age-related and gender-specific prevalence trends. Notably, excess weight and a higher BMI highlight the role of obesity as a significant OA risk factor. Bilateral knee involvement is common, with a strong link between OA and obesity. This research underscores the need for further longitudinal investigations, considering a broader range of potential influencing factors, to better understand the multifaceted nature of knee OA in the context of obesity in Saudi Arabia.
